# Co-administration of Four-Factor Prothrombin Complex Concentrate With Andexanet alfa for Reversal of Nontraumatic Intracranial Hemorrhage

**DOI:** 10.1177/00185787241229192

**Published:** 2024-02-16

**Authors:** Sophia Pathan

**Affiliations:** 1 Hospital of the University of Pennsylvania, Philadelphia, PA, USA

**Keywords:** 4F-PCC prothrombin complex concentrate, andexanet, anticoagulants, intracranial bleeding, reversal, thromboembolism, neurocritical care

## Abstract

**Objective:** Andexanet alfa is approved for the reversal of life-threatening or uncontrolled bleeding due to factor-Xa inhibitors. Data are limited on outcomes for patients who receive both andexanet alfa and 4-factor prothrombin complex concentrate (4F-PCC). The aim of this case series is to evaluate the safety and efficacy outcomes in patients receiving the two agents in combination. **Methods:** Electronic medical records of patients who received both 4F-PCC and andexanet alfa for nontraumatic intracranial hemorrhage from January 2019 to March 2022 were retrospectively reviewed. Hemostatic efficacy and complications related to concurrent use of 4F-PCC with andexanet alfa were documented. **Results:** Nine patients received 4F-PCC and andexanet alfa for reversal of factor Xa inhibitor-associated intracranial bleeding, eight of whom required reversal of apixaban. Of these nine patients, five patients died within 28 days for a 56% incidence of mortality. The average time from 4F-PCC administration to andexanet alfa administration was 3 hours and 9 minutes. Most doses of andexanet alfa were given for concern for bleed expansion after 4F-PCC administration. Hemostatic efficacy based on stability of repeat computed tomography scans post-administration of both agents was found in six patients (66.67%), with a 55.56% n incidence of thromboembolism, including two pulmonary embolisms, two deep vein thromboses, and one renal artery thrombosis. **Conclusion**: Risks and benefits should be weighed to determine if there is benefit to adding andexanet alfa to 4F-PCC in patients with incomplete hemostasis and life-threatening hemorrhage. The combination of andexanet alfa and 4F-PCC may increase the risk of thrombotic complications without improving mortality.

## Introduction

The factor Xa inhibitors apixaban and rivaroxaban are anticoagulants used in the treatment and prevention of thrombotic events and are often recommended over vitamin K antagonists due to a more favorable side effect profile.^1,2^ With increasing utilization of the factor Xa inhibitors, managing severe major bleeding is essential, as these agents have a rate of intracranial hemorrhage (ICH) of 0.1% to 0.2%.^3,4^ Antithrombotic-associated intracranial hemorrhage (ICH) can be devastating, so use of a reversal agent targeted toward anti-factor Xa effects is critical in the restoration of hemostasis to improve patient outcomes. Reversal agents can also be used with the intention of reversing factor Xa inhibitors prior to urgent surgical procedures, such as neurosurgery.^5^

Prior to the May 2018 approval of andexanet alfa by the Food and Drug Administration, patients treated with apixaban or rivaroxaban did not have a reversal agent targeted to their specific anti-factor Xa effects. Patients with factor Xa inhibitor-associated ICH were managed with supportive care and administration of a prothrombin complex concentrate (PCC) such as a four-factor PCC (4F-PCC) or an activated PCC (aPCC).^4^ PCCs contain variable amounts of factors II, VII, IX, and X, as well as proteins C and S, with dosing based on their factor IX component.^6^ The benefits of PCCs include their increase in the levels of circulating clotting factors and increased thrombin generation, but disadvantages include the associated thrombotic risks of providing clotting factors exogenously.^7 [Bibr bibr8-00185787241229192]^

In contrast to 4F-PCC, inactivated-zhzo or andexanet alfa is a modified recombinant decoy protein of human factor Xa designed specifically to bind and sequester factor Xa inhibitor molecules like apixaban and rivaroxaban. By binding the factor Xa inhibitor medications, andexanet alfa can rapidly reduce anti-factor Xa activity.^9,10^ Andexanet alfa can therefore reverse factor Xa inhibition and restore endogenous thrombin activity.^11^ The increased thrombin activity relates to the increased pro-coagulant risks of andexanet alfa, so the package insert carries a boxed warning for the risk of arterial and venous thromboembolic events, ischemic risks, cardiac arrest, and sudden death.^11^

Data is currently inconclusive regarding whether 4F-PCC or andexanet alfa is preferred in patients with factor Xa inhibitor-associated bleeding. Numerous studies have been published with conflicting data on whether one agent or the other is more efficacious.^12-18^ Retrospective and prospective studies have demonstrated the efficacy of 4F-PCC in treating factor Xa inhibitor-associated bleeding events with acceptable rates of adverse events.^19,20^ In addition, there is an ongoing, randomized controlled trial comparing 4F-PCC and andexanet alfa in ICH patients.^21^

Still, there is little data or guidance regarding concomitant use of 4F-PCC and andexanet alfa, with most information coming from case reports and case series.^22-27^ By far the largest concern of giving these agents in combination is the increased risk of thrombosis. The meta-analysis by Nederpelt et al. suggests the risk of thrombosis with 4F-PCC could range from 0% to 9.1%, and the risk with andexanet alfa could range from 2.9% to 16.7%.^23^ For the combination of these two agents, the risk could be much higher. In fact, the most recent of such case series by Liu et al. pooled thromboembolism incidence from previous studies and found that the combination of 4F-PCC and andexanet alfa led to an incidence of thromboembolism of 35%.^28^ Another recent case series by Bradshaw et al. found that 2 of 5 patients (40%) experienced thromboembolism.^29^

This analysis examines patients who received both 4F-PCC and andexanet alfa for reversal of factor Xa inhibitor-associated nontraumatic intracranial bleeding at a large academic medical center, with the goal of defining safety and efficacy outcomes.

## Methods

This retrospective case series included all patients who required reversal of an oral factor Xa inhibitor with andexanet alfa after receiving 4F-PCC for an intracranial bleed. This was a convenience sample of patients from January 2019 to March 2022 and featured mainly apixaban use but evaluated all patients who received apixaban or rivaroxaban. Patients were excluded if their primary bleed was not neurologic in nature or if it was traumatic in nature. The electronic medical record was utilized to identify patients who received andexanet alfa and 4F-PCC in the adult neurocritical care unit.

At this institution, there is no restriction criteria for use of andexanet alfa or 4F-PCC as long as the patient received apixaban or rivaroxaban in the prior 24 hours and presented with intracranial bleeding. However, any repeat doses require hematology approval, and after andexanet alfa administration, a hematology consult is required. Cases of using both andexanet alfa and 4F-PCC are not delinieated in institutional guidelines but theoretically would require hematology consult as a “repeat dose.”

Additionally, at this institution, anti-Xa levels are calibrated for specific agents, with levels for unfractionated heparin, enoxaparin, apixaban, and rivaroxaban available in-house. Anti-Xa levels to indicate factor Xa inhibitor presence are not required prior to administration of andexanet alfa. Dosing of 4F-PCC is at the discretion of the provider, with no specific guidance outlining whether fixed or weight-based dosing should be utilized. There is no guidance in choice of either andexanet alfa or 4F-PCC at this institution, with choice of agent and dose at the discretion of the covering provider.

Patients experiencing multiple foci of bleeding were included in the analysis, but hemostatic efficacy was defined by no expansion of the bleed on post-reversal imaging, in contrast to the ANNEXA-4 hemostatic efficacy examination criteria.^30^ This was based on initial radiologist impression, as this is what guided therapy at the time. The goal was to conduct a safety analysis evaluating thromboembolic events within 30 days or prior to hospital discharge. If patients were no longer admitted for the 30 day period, rehabilitation or clinic notes were utilized for information. The rehabilitation facilities and clinics are part of the health system, so documentation was considered accurate. If this information was not available, then the patient’s last known outcomes were considered.

Collected data included but was not limited to patient demographics, past medical history, pertinent laboratory values, anticoagulation therapy, admission information, and discharge information. Time stamps during patient assessment were collected, starting with the time head CT scan was ordered through the time andexanet alfa was administered, to estimate the mean time between each step from ICH recognition to oral factor Xa inhibitor reversal. If data were not available for each time point, then the next closest time point was used.

This review used a sample of data following approval of andexanet alfa and standardization of the ordering process. Sample size was determined by pulling data on all eligible patients in the time period of the review. Given the small sample size and lack of comparator groups, descriptive statistics were used to summarize the collected data. Data were analyzed using Microsoft Excel (2017).

## Cases

### Case 1

Patient 1 was a 69-year-old male with a past medical history of multiple strokes with residual right hemiparesis, hypertension, type 2 diabetes mellitus, recurrent urinary tract infections, heart failure, and recent deep vein thrombosis for which he was on apixaban with the last dose about 12 hours before presentation. On presentation, his Glasgow Coma Scale (GCS) was 14, with a supratentorial ICH volume of 18 cm^3^ and no IVH (ICH score 1). He presented from an outside hospital after reversal with 4F-PCC 50 units/kg (4,650 units) . His head CT at the outside hospital also showed local edema of about 2.2 cm at its widest spanning about four slices.

On arrival, the decision was made to give andexanet alfa without another CT scan due to concern for worsening exam. Coagulation labs at this time showed a normal INR of 1.1 and normal aPTT of 36.3 seconds. Repeat CT was done two hours after andexanet administration. This scan read “Hyperdensity overlying the right thalamus measuring 2.7 × 1.8 cm in keeping with clinically suspected right thalamic hemorrhage.”

After administration of both agents, the patient’s repeat CT scans and MRIs were all stable. No neurosurgical interventions were made. The patient was transferred from the neuro ICU to the step-down unit within 12 hours of andexanet alfa administration and was restarted on prophylactic anticoagulation within 48 hours, with the decision on restarting full anticoagulation to be made outpatient at a follow-up anticoagulation clinic. He was ultimately discharged after 10 days in the hospital to a skilled nursing home.

### Case 2

Patient 2 was an 84-year-old male with a past medical history of heart failure, myocardial infarction, peripheral artery disease, and atrial fibrillation with a pacer (CHA2DS2-VASc 5) for which he was on rivaroxaban. He presented to an outside hospital with a Hunt Hess 5 (coma, decerebrate rigidity) modified Fisher 4 (thick subarachnoid bleed with intraventricular hemorrhage) subarachnoid hemorrhage, as well as hemorrhage in the prepontine cistern with displacement of the lower pons and medulla posteriorly by an approximately 2 cm hematoma. Additionally, there was subarachnoid hemorrhage extending superiorly into the suprasellar cistern and there was intraventricular hemorrhage within the third and fourth ventricles. The patient received 52 units/kg of 4F-PCC (approximately 4,000 units) at the outside hospital and was transferred to this institution.

Hematology was consulted o upon the patient’s arrival and recommended andexanet alfa at a low dose as it was unclear if there was ongoing bleeding and the patient would need an external ventricular drain (EVD) placed. At this time, the patient’s aPTT was 34.9 seconds and INR was 1.2, and the patient received the andexanet alfa dose and underwent EVD placement. The repeat head CT after andexanet alfa administration showed new or worsening bleeding, and the patient’s EVD was deemed nonfunctional. At this time, the family decided to change the patient’s code status to “Do Not Resuscitate,” after which he was transitioned to comfort care and passed away after three days of admission.

### Case 3

Patient 3 was an 82-year-old male with a past medical history of hyperlipidemia, hypertension, chronic obstructive pulmonary disease (with a recent hospitalization for an exacerbation), and paroxysmal atrial fibrillation, for which he was on apixaban with a CHA2DS2-VASc of 4. He presented to an outside hospital with an acute right cerebellar intraparenchymal hemorrhage with intraventricular extension (ICH score 3), subarachnoid hemorrhage, and mild hydrocephalus. A CT angiogram was done at the outside hospital with findings including venous varices throughout the surface of the bilateral cerebral hemispheres with the largest in the inferolateral posterior right cerebellar hemisphere adjacent to the intraparenchymal hemorrhage, corresponding with a ruptured arteriovenous malformation (AVM). Due to this, the patient received a flat 1,430 units of 4F-PCC for reversal (approximately 17.29 units/kg), had a right frontal EVD placed, and then was transferred to this institution.

At this time, his aPTT was 33.4 seconds and his INR was 1.1 seconds, and hematology was consulted and recommended using andexanet alfa to further reverse the patient’s apixaban due to “the greater body of evidence for andexanet alfa than 4F-PCC for reversal of apixaban in patients with intracranial hemorrhage, as well as the critical nature of the patient clinically” as well as the repeat CT scan showing a mild interval increase in both the intraventricular and right cerebellar hemorrhages. The low dose of andexanet alfa was used due to the dose of apixaban the patient was receiving, as well as the time of the last dose. Afterward, the patient’s scans showed stable hemorrhages. The patient’s AVM was deemed inoperable secondary to its location, and the patient remained in the ICU for delirium management and vasodilatory shock as well as new pulmonary embolism. The patient was transferred to an inpatient rehabilitation facility after 17 days in the ICU and six days in the step-down unit.

### Case 4

Patient 4 was a 67-year-old female with a past medical history of hypertension, DVT (on apixaban) and right PCA/SCA aneurysm post-stent-assisted coil with multiple re-stenting procedures (most recently three months prior, on dual antiplatelet therapy with aspirin and clopidogrel). She presented to an outside hospital with a Hunt Hess 3 (mild focal deficit) modified Fisher 2 (thin SAH with IVH) subarachnoid hemorrhage in the pre-pontine and perimesencephalic regions, along with a small intraventricular hemorrhage with hydrocephalus. She was reversed with desmopressin and 4F-PCC 25 units/kg (~2,000 units) before being transferred to this institution. At this time, her aPTT was 24.8 seconds and her INR was 1.

At this hospital, she was further reversed with low dose andexanet alfa for EVD placement after consultation with hematology. She was also given two units of platelets for reversal of her antiplatelet agents. After EVD placement, she went to the operating room for cerebral angiography and no interventions were made. She also redemonstrated residual filling at the inferior aspect of her previously treated right PCA-SCA aneurysm which now measured approximately 20 mm × 8 mm, increased from her scan 3 months before.

She was restarted on prophylactic anticoagulation at 48 hours, with no intentions of restarting either her apixaban or her clopidogrel. Her aspirin was restarted on day 7 of admission. Her course was complicated by cerebrospinal fluid leak, urinary tract infections, and new DVT, cerebral salt wasting, but she was discharged with a left frontal ventricular-peritoneal shunt to a rehabilitation facility after 20 days in the ICU and 24 days in the hospital total.

### Case 5

Patient 5 was a 74-year-old female with a past medical history of human immunodeficiency virus, prior left frontal stroke, atrial fibrillation (CHA2DS2-VASc 4, on apixaban), who presented to an outside hospital after being found unresponsive at her facility. The head CT demonstrated a large right temporal intraparenchymal hemorrhage with sylvian Hunt Hess 4 (stupor with motor deficits and intermittent reflex posturing) modified Fisher 4 (thick SAH with IVH) subarachnoid hemorrhage, intraventricular hemorrhage (ICH score 4), and midline shift. The patient received about 4,000 units (53.55 units/kg) of 4F-PCC for her apixaban reversal and was intubated prior to transfer for further management.

At this institution, her INR was 1.3 and her aPTT was 34.1 seconds, and hematology was consulted. Hematology recommended against giving andexanet alfa. However, neurosurgery ordered and administered a low dose in the operating room, where she underwent a right decompressive hemicraniectomy and clot evacuation with an intraoperative left frontal EVD placement. The patient’s course was complicated by atrial fibrillation with rapid ventricular rate, comatose state, respiratory failure, pneumonia, and cerebral edema. She remained in the hospital for 13 days before being transitioned to comfort measures only and passing away.

### Case 6

Patient 6 was a 78-year-old male with a past medical history of biphasic mesothelioma complicated by malignant left pleural effusion post-video-assisted thoracoscopic surgery pleurodesis two months prior, coronary artery disease with history of percutaneous coronary intervention, and atrial fibrillation (CHA2DS2-VASc 4) on apixaban. He presented with blurry vision and word finding difficulty and was found to have a right cerebellar intracranial hemorrhage with mass effect at the fourth ventricle (ICH score 2) and left frontal subarachnoid hemorrhage. Neurosurgery said there was no acute intervention to make, and the patient’s apixaban was reversed with the high dose andexanet alfa protocol as well as with about 50 units/kg 4F-PCC (3,737 units), both given at the same time. Based on the patient’s apixaban dose and timing, he should have received the low andexanet alfa dose rather than the high dose.

Later that day, the patient experienced dyspnea requiring intubation and escalation of care to the ICU, followed by cardiac arrest that was determined to be secondary to a pulmonary embolism. The patient underwent four rounds of chest compressions and epinephrine, and the family chose to withdraw support and the patient expired.

### Case 7

Patient 7 was a 78-year-old female with a past medical history of metastatic breast cancer and prior DVTs, so the patient was on apixaban. She presented to the hospital with a right M2 occlusion for which she did not undergo thrombolysis secondary to her apixaban usage but did undergo a mechanical thrombectomy with TICI2a reperfusion. Her course was complicated by hemorrhagic conversion of her stroke, leading to reversal of her apixaban using 1,587 units of 4F-PCC (~38.2 units/kg). At this time, her aPTT was 32.3 seconds and her INR was 1.3. An hour later, hematology was consulted for andexanet alfa use, as the repeat head CT after reversal with 4F-PCC showed increased extent of bleed with midline shift and mass effect. Hematology discussed the theoretical increased risk of thrombosis with both PCC and andexanet alfa. However, given the clinical scenario, this risk was considered outweighed by the risk of worsening hemorrhage, and she was reversed with low dose andexanet alfa.

Two days later, she was found to have right-sided internal jugular vein thromboses with filling defects. At this time, she was started on heparin subcutaneously as prophylaxis and transferred to the floor for further management. On the floor, her course was complicated by a urinary tract infection, new internal jugular thrombosis, and right temporal intermittent rhythmic delta activity, for which she was started on levetiracetam prophylaxis. She was discharged after 25 days in the hospital with follow-up clinic instructions to determine the start date for full anticoagulation.

### Case 8

Patient 8 was a 78-year-old male with a past medical history of osteoarthritis with left knee replacement, hypertension, metastatic prostate cancer, and atrial fibrillation (CHA2DS2-VASc 3) for which he was previously on rivaroxaban but his course had been complicated by a large left occipital and small left cerebellar stroke, so he was transitioned to apixaban. He presented to an outside hospital with a left occipital and left cerebellar intraparenchymal hemorrhage where he received reversal with 4F-PCC at 50 units/kg and was transferred to this institution.

During his follow up head CT, he had an evolving left occipital lobe hemorrhage and a new 5 mm intraparenchymal hemorrhage, prompting the team to order another 25 units/kg of 4F-PCC, bringing his total 4F-PCC to about 72 units/kg within the day. Of note, this practice is not common and is not recommended by the package insert, as the safety and effectiveness of repeat dosing above the recommended 50 units/kg weight-based dose have not been established.^6^ The risk of thrombosis with repeat administration of concentrated factor product often can outweigh the benefit of additional factor given, particularly as there is data demonstrating use of fixed 4F-PCC dosing as noninferior to weight-based dosing.^31^

In addition to the 4F-PCC, this patient also received 1 unit of platelets. At this time, his INR was 2 and his aPTT was 33.2 seconds. After this, neurosurgery was consulted for a decompressive hemicraniectomy and EVD placement and requested low dose andexanet alfa. The patient received the andexanet alfa in the operating room prior to the procedure and received 2 units of platelets, 1 unit of fresh frozen plasma, 1 unit of packed red blood cells, and 2.8 L crystalloids during the procedure.

Post-operatively, the patient’s head CT showed increased multicompartmental hemorrhages and new trace petechial hemorrhages. The patient was also found to have microvascular thromboses leading to cool extremities and renal thromboses leading to acute kidney injury. The family decided to transition the patient to comfort measures only and the patient was terminally extubated after four days in the ICU.

### Case 9

Patient 9 was a 79-year-old male with a past medical history of prior cerebellar stroke on apixaban for patent foramen ovale, hypothyroidism, polymyalgia rheumatica, hypertension, and hyperlipidemia. He presented to the oncology unit with new diagnosis of acute myeloid leukemia, was admitted for hyperleukocytosis and experienced an acute mental status change during an apheresis session. At this time, his platelet count was 55,000, his INR was 4.3, and his aPTT was 44.1 seconds. Due to the mental status change, he had a head CT and was found to have a multifocal intraparenchymal hemorrhage with the largest bleed located in the right frontal lobe, measuring 2.9 × 1.8 cm. His ICH score was 1.

He was given 2,585 units (34 units/kg) of 4F-PCC about 10 minutes before low dose andexanet alfa and also received 1 unit of cryoprecipitate and 3 units of platelets. He was then transferred to the intensive care unit. Repeat CT head 6 hours later showed stable hemorrhages. His blood pressure initially improved after intubation, but he then developed progressive shock refractory to fluids and increased vasopressor doses despite escalation to three vasopressors. His cardiac echo was remarkable for severely dilated right ventricle and moderate to severely decreased right ventricular function with severely elevated pulmonary arterial systolic pressure. Given his overall clinical picture, it was felt that this was a non-recoverable event. In discussion with his family, he was transitioned to comfort care and was terminally extubated that afternoon.

## Results

Over a 39-month period, thirteen patients received andexanet alfa and 4F-PCC at the study institution. Of these thirteen, nine patients received the combination for ICH, three patients received the combination after a cardiac surgical procedure, and one patient received it for massive spontaneous hemoptysis in the medical intensive care unit. Only nine patients were included in this analysis. Of the nine patients, six cases (66.67%) showed potential hemostasis after the combination of andexanet and 4F-PCC, a value lower than what is reported in the ANNEXA-4 trial for andexanet alfa alone.^12,30^

### Baseline Characteristics

Baseline characteristics can be found in Table 1. The mean age of all patients was 76.56 ± 5.61 years, with most patients being white males. Estimated creatinine clearance varied between patients, with a mean of 79.35 mL/min ± 44.09 mL/min. Mortality occurred in five patient cases. Further adverse events are detailed in Table 2.

**Table 1.** Baseline Demographics.

Category	Result (n = 9)
Age in years, mean ± SD	76.56 ± 5.61
Male sex, n (%)	6 (66.67%)
White race, n (%)	5 (55.56%)
Height (cm), mean ± SD	169.2 ± 7.97
Weight (kg), mean ± SD	74.37 ± 13.6
BMI (kg/m^2^), mean ± SD	26.1 ± 5.56
GCS Score, mean ± SD	9.90 ± 5.13
SOFA Score, mean ± SD	3.44 ± 3.68
aPTT prior to andexanet administration (seconds), mean ± SD	32.59 ± 5.97
INR prior to andexanet administration, mean ± SD	1.71 ± 0.99
aPTT 12 hours after andexanet administration (seconds), mean ± SD	32.66 ± 4.72
INR 12 hours after andexanet administration, mean ± SD	2.42 ± 2.17
Platelets prior to anexanet administration (× 10^3^/µL), mean ± SD	171 ± 116
Length of stay (days), mean ± SD	11.78 ± 9.9
ICU Length of stay (days), mean ± SD	7.33 ± 7.25
Subarachnoid bleed, n (%)	5 (55.56%)
Multiple foci of bleeding, n (%)	7 (77.78%)
Additional blood products received, n (%)	4 (44.44%)
Intubated, n (%)	4 (44.44%)
Use of high dose andexanet alfa, n (%)	1 (11.1%)
Neurosurgical procedure performed (excluding EVD placement), n (%)	2 (22.22%)
Mortality	5 (55.56%)

*Note*. SD = standard deviation; BMI = Body Mass Index; SOFA = Sequential Organ Failure Assessment; ICU = intensive care unit; EVD = external ventricular drain.

**Table 2.** Adverse Events.

Category	Result (n = 9)
Death within 48 hours	3 (33.33%)
Death within 30 days	5 (55.56%)
Bleeding event within 30 days	1 (11.11%)
Thrombotic event within 30 days	5 (55.56%)

Indications for pre-admission factor Xa inhibitor use were atrial fibrillation (55.56%) and active thrombus (44.44%), with no concerning drug interactions in each case. Five of the nine patients (55.56%) had a prior history of strokes before presentation, with eight of the nine patients (88.89%) presenting on apixaban. All apixaban patients were on the standard 5 mg twice daily dosing, and the rivaroxaban patient was on 20 mg daily dosing. This was appropriate dosing for all patients. Further information is detailed in Table 3.

**Table 3.** Patient Case Data.

	Case 1	Case 2	Case 3	Case 4	Case 5	Case 6	Case 7	Case 8	Case 9
FXa inhibitor	Apixaban 5 mg q12h	Rivaroxaban 20 mg daily	Apixaban 5 mg q12h	Apixaban 5 mg q12h	Apixaban 5 mg q12h	Apixaban 5 mg q12h	Apixaban 5 mg q12h	Apixaban 5 mg q12h	Apixaban 5 mg q12h
Indication for anticoagulation	DVT	Atrial fibrillation	Atrial fibrillation	DVT	Atrial fibrillation	Atrial fibrillation	DVT	Atrial fibrillation	Thrombus in patent foramen ovale
SOFA Score prior to 4F-PCC administration	1	10	3	0	4	2	0	2	9
NIHSS baseline	11	-	1	16	-	1	7	-	-
Platelets prior to andexanet-alfa dose (/µL)	470,000	159,000	129,000	191,000	222,000	84,000	135,000	96,000	55,000
Time of last Xa inhibitor dose	Unknown (likely <8 hours)	Unknown (likely >8 hours)	6 hours prior to 4F-PCC admin	9 hours prior to 4F-PCC admin	Unknown (likely <8 hours)	5 hours prior to 4F-PCC admin	14 hours prior to 4F-PCC admin	25 hours prior to 4F-PCC admin	14 hours prior to 4F-PCC admin
Indication for reversal	R thalamic bleed	SAH	Cerebellar ICH with IVH, SAH 2/2 AVM rupture	SAH in pre-pontine and peri-mesencephalic regions, small IVH	R temporal IPH with sylvian SAH, IVH	R cerebellar ICH and left frontal SAH	IPH conversion s/p thrombectomy of R M2 occlusion	L occipital and L cerebellar IPH	Multifocal IPH
Andexanet alfa dose	Low	Low	Low	Low	Low	High	Low	Low	Low
4F-PCC dose	4,650 units (50 units/kg)	4,000 units (52 units/kg)	1,430 units (17.29 units/kg)	2,000 units (23.81 units/kg)	4,000 units (53.55 units/kg)	3,737 units (49.63 units/kg)	1,587 units (38.52 units/kg)	5,285 units (72.9 units/kg)	2,585 units (34 units/kg)
First product given at outside hospital	Yes	Yes	Yes	Yes	Yes	No	No	Yes	No
Additional blood products received	-	-	-	2 units platelets	-	2 units platelets, 5 units cryo-precipitate, 1 unit pRBCs	-	2 units plasma, 4 units platelets, 1 units pRBCs	2 units plasma, 2 units platelets, 5 units cryo-precipitate, 1 unit pRBCs
Thrombotic complications	-	-	Pulmonary embolism	DVT	-	Pulmonary embolism	Internal jugular vein thrombus	Renal artery thrombosis	-
Anticoagulation restart	48 hours, prophylaxis	-	96 hours, prophylaxis	48 hours, prophylaxis	48 hours, prophylaxis	-	48 hours, prophylaxis	-	-
Disposition	Skilled nursing facility	Morgue	Rehab	Rehab	Morgue	Morgue	Skilled nursing facility	Morgue	Morgue

All patients presented to the hospital with ICH, and six patients received 4F-PCC at an outside hospital (66.67%) with the remaining three patients (33.33%) receiving both the 4F-PCC and andexanet alfa at the study hospital. All patients received 4F-PCC first, followed by andexanet alfa. Five of the nine patients presented with subarachnoid hemorrhage (55.56%), and seven of the nine patients (77.78%) had multiple foci of bleeding.

Per the institutional guideline, all patients received andexanet alfa for an appropriate indication. As all but one patient were on apixaban 5 mg, those patients should have all received the low dose andexanet alfa. One patient received the high dose, which was inappropriate, and this patient experienced a pulmonary embolism and passed away. The patient on rivaroxaban 20 mg received the appropriate dose of andexanet alfa based on prior dose timing. As the guidelines do not specify when to use both agents in conjunction, practice is to dose as though 4F-PCC had not been given, so all but one of the patients received an appropriate dose of andexanet alfa. Against the institutional guidelines, four of the nine patients did not receive a hematology consult (44.44%).

Most patients had low SOFA scores prior to 4F-PCC administration, with a mean SOFA score of 3.44 ± 3.68. Though the mean SOFA scores were low, these patients were unstable due to their intracranial bleeds and based on their head CT scans. Average Glasgow Coma Scale (GCS) on arrival to the study institution was 9.89 ± 5.13, and three of the patients had scores less than 8 (33.33%).

The average international normalized ratio (INR) after 4F-PCC and prior to andexanet alfa administration was 1.96 ± 0.96, likely elevated due to DOAC use and patient instability. Twelve hours after administration of both agents, the average INR was 2.42 ± 2.17. Non-heparin anti-Xa levels are not readily available at the study institution, so although the International Society on Thrombosis and Haemostasis recommends consideration of DOAC reversal for patients with serious bleeding and a DOAC level > 50 ng/mL, this was only evaluated in one patient, who had an apixaban anti-Xa level of 151.95 and qualified for reversal.^13^

The average time from head CT to administration of 4F-PCC was 1 hour and 16 minutes, and the average time from 4F-PCC administration to andexanet alfa administration was 3 hours and 9 minutes. Exact times between head CT and reversal agent doses are available in Table 4. Four of nine patients (44.44%) received additional blood products, and two of nine patients (22.22%) went to the operating room for a decompressive hemicraniectomy. Of note, rates of EVD placement at this institution may be higher than other institutions, and andexanet alfa tends to be this institution’s preferred agent prior to neurosurgical procedures and EVD placements.

**Table 4.** Computed Tomography Scan Information.

Case	CT prior to 4F-PCC admin	Time between head CT and 4F-PCC (hours)	Time between 4F-PCC and andexanet (hours)	Time between original head CT and andexanet (hours)	Andexanet relation to repeat head CT	Repeat CT after 4F-PCC admin (between 4F-PCC and andexanet)	CT at least 12 hours after andexanet administration
1	R thalamus IPH (2.3 cm × 1.9 cm × 2.1 cm)	1:55	1:50	3:45	Before	Re-demonstrated right thalamic parenchymal hematoma with local mass effect and partial effacement of the lateral and third ventricles.	
2	SAH of prepontine and suprasellar cisterns IVH within third and fourth ventricles Minimal hemorrhages layering in the occipital horns bilaterally	1:33	2:51	4:24	Before	New or increased intracranial or intraspinal hemorrhage	
3	R cerebellar IPH (2.5 cm × 3.8 cm diameter), with breakthrough IVH and moderate subacute blood products in the lateral, third, and fourth ventricles	0:31	9:42	10:13	After	Mild interval increase in extensive IVH. Mild interval increase in R ICH.	Stable extensive IVH. Stable R cerebellar hemorrhage
4	Small prepontine and perimesencephalic SAHs Small IVHs with hydrocephalus	0:25	4:25	4:50	Before	Unchanged aSAH in the basal cisterns.	Evolving areas of SAH and IVH.
5	Significant right-sided acute intracranial hemorrhage involving the subarachnoid spaces of the right lateral ventricle and right sylvian fissure. There is a more masslike area of hemorrhage in the right temporal lobe which is likely at least partially intraparenchymal. Surrounding mass effect with 9 mm of leftward midline shift.	0:40	5:59	6:39	After	Ganglia region is increased in size from when there was a small amount of intraventricular extension into the R lateral ventricle, now extending through the entire ventricular system and into the craniocervical junction.	Similar appearance of multicompartmental ICH.
6	Large acute intraparenchymal hemorrhages in the cerebellar hemispheres, greater on the right. Small volume subarachnoid hemorrhage within the left frontal sulci.	1:10	0:01	1:11	Before	Stable IPHs. Stable L frontal SAH.	
7	Scan concerning for increased hemorrhage. The hematoma measures approximately 3.7 cm hematoma	1:53	1:38	3:31	Before	Interval decrease in the R perisylvian IPH with decreased mass effect and midline shift.	Unchanged evolving R sylvian/perisylvian multicompartmental hemorrhage.
8	Acute L occipital hemorrhage concerning for hemorrhagic transformation of acute on chronic ischemic change New IPH within the cerebellar hemispheres L > R L frontal lobe punctate focus of IPH	1:46	1:47	3:33	Before	Evolving L occipital lobe hemorrhagic conversion. New linear punctate densities in the R parietal lobe, may represent new foci of hemorrhage. New 5 mm IPH in the L occipital lobe. Interval increase in the L frontal lobe IPH. Interval development of IVH within the occipital horns.	Increased multicompartment supratentorial multicompartment hemorrhage with new hemorrhage along the R frontal catheter tract, SDHs along the tentorial leaflets, and likely new trace petechial hemorrhage in the R occipital infarct.
9	New or increased ICH or intraspinal hemorrhage Innumerable scattered bilateral IPHs	1:39	0:10	1:49	Before	Stable numerous bilateral cerebral hematomas, with mild surrounding vasogenic edema. No definite new or increasing hemorrhage. No midline shift or herniation.	No definite new or increasing hemorrhage. No midline shift or herniation.

*Note*. R = right; IPH = intraparenchymal hemorrhage; SAH = subarachnoid hemorrhage; aSAH = aneurysmal subarachnoid hemorrhage; IVH = intraventricular hemorrhage; ICH = intracranial hemorrhage; L = left; SDH = subdural hemorrhage.

### Outcomes

Effective hemostasis within 12 hours of andexanet alfa administration after 4F-PCC could not be established based on ANNEXA-4 criteria, as seven patients experienced bleeding in multiple brain foci and CT scan data did not report bleed sizes to estimate percent changes. However, andexanet alfa was given before stability head CT scans in seven of the nine cases (77.78%), and in three cases (33.33%) the repeat scans showed continued bleeding directly after andexanet administration. In these cases, clinical judgement without objective data was used to make the call to use andexanet alfa after (or with) 4F-PCC. Of the seven cases where andexanet alfa was given without repeat scan, four patients experienced a thrombotic event.

Five of the nine patients (55.56%) experienced mortality within 30 days, and of these patients, three experienced mortality within 48 hours (33.33%). This was significantly higher than the ANNEXA-4 rate of 14%. Of the five patients who experienced mortality, three patients experienced mortality secondary to their intracranial bleeds, one patient experienced mortality due to a thrombosis from the andexanet alfa and 4F-PCC combination, and one patient experienced mortality due to likely cardiogenic shock. This is detailed in Table 2.

As most 4F-PCC doses were given at outside hospitals, it was not standardized as to whether patients were being given fixed doses or weight-based doses. Two of the nine cases (22.22%) received doses under what would be considered the standard fixed dose of 2,000 units. Five of the nine cases (55.56%) received at least the standard weight-based dose of 50 units/kg. One patient received higher than the weight-based recommended dose. There was no difference in outcomes based on location of original 4F-PCC dose at outside hospital versus this institution.

Prophylactic anticoagulation with subcutaneous heparin was started within 48 hours for four of the nine patients (44.44%), with a fifth patient starting prophylactic subcutaneous heparin with 96 hours. Five patients experienced a thrombotic event within 30 days, for a thrombosis risk of 55.56%.

## Discussion

The ANNEXA-4 trial brought andexanet alfa to the forefront of factor Xa inhibitor reversal, but the study excluded patients who received any PCC within the seven days prior to screening. Considering the lack of data of the two agents in combination, this retrospective evaluation adds to current case series data evaluating patients who received andexanet alfa in combination with 4F-PCC for reversal of factor Xa inhibitor-associated intracranial bleeding.

4F-PCC has been shown to effectively restore hemostasis in factor-Xa inhibitor-related hemorrhage through elevation of coagulation factor levels and counteraction of factor-Xa inhibitor anticoagulation effects.^31-34^ Most data are retrospective, so patient baseline characteristics vary and do not lead to exclusion from studies. In a recent meta-analysis by Chiasakul et al. comparing fixed and weight-based 4F-PCC dosing, hemostatic effectiveness of 4F-PCC in factor-Xa inhibitor reversal was 81.0% for weight-based or variable dosing in comparison with 72.9% in the single fixed dosing study being analyzed, though there was moderate to high heterogeneity between studies. In these patients, thromboembolic event rates were about 4%, with mortality at 28.5%.^31^ In contrast, this retrospective case series of patients specifically had low hemostatic effectiveness compared to prior data, motivating the consideration of andexanet alfa after adequate 4F-PCC dosing.

With low GCS baselines, need for surgical procedures, low survival likelihood, multiple foci of brain bleeding, and administration of other factors or blood products (such as 4F-PCC), this patient population does not mirror the ANNEXA-4 population. Further comparisons between ANNEXA-4 and this retrospective case series population can be seen in Table 5. As this patient population did not mirror ANNEXA-4, this analysis too could not define hemostatic efficacy in the same way as ANNEXA-4, where patients achieved good or excellent hemostasis based on hemorrhage volume expansion from pre-andexanet alfa administration volume. In real practice, there are many patients with neurologic injuries fitting criteria outside of the ANNEXA-4 trial, and more studies are needed to fully understand the impact of one or both agents on patient outcomes.

**Table 5.** ANNEXA-4 Compared to This Case Series.

	ANNEXA-4		Case series
Baseline characteristics	Exclusion Criteria • Surgery within 12 hours after presentation • Intracranial hemorrhage with GCS < 7 • Estimated intracerebral hematoma volume >60 mL • Expected survival <1 month • Major thrombotic event within prior 2 weeks • One of the following within 7 days prior to screening: vitamin K antagonist, dabigatran, prothrombin complex concentrate, or whole blood or plasma Most patients received apixaban or rivaroxaban for mainly atrial fibrillation and/or VTE. Adapted from ANNEXA-4:		This case series included patients that would have fallen into the exclusion criteria of ANNEXA-4, so the populations do not correspond. • 22.22% of patients underwent a neurosurgical procedure • 44.44% of patients received additional blood products either before or after andexanet (with 100% of patients receiving 4F-PCC in addition to the andexanet) • 55.56% of patients in this series were receiving anticoagulation for atrial fibrillation, with the remaining 4 patients receiving anticoagulation for active thrombus • Mean GCS in this population was 9.9 ± 5.13, which is a wider range than ANNEXA-4 but is also lower than the ANNEXA-4 baseline • Most patients in this case series had multiple foci of bleeding (77.78%) including subarachnoid bleeds (55.56%) • This retrospective series did not include traumatic bleeds, which were included in ANNEXA-4 • The time from baseline scan to andexanet was a lot longer in this cohort compared to ANNEXA-4 because 4F-PCC was administered first in all cases
	ICH patient characteristics (N = 227)	Analysis	
	GCS, mean	14	
	Time from baseline scan to andexanet, hours, mean ± SD	2.37 ± 1.61	
	Multi-compartment bleeding, n (%)	69 (30.4)	
	Intracerebral site, n (%)	137 (60.35)	
	Subdural, n (%)	75 (33.04)	
	Subarachnoid, n (%)	57 (25.11)	
Results	• 80% ICH patients with “excellent” or “good” hemostatic efficacy at 12 hours • For intracerebral hemorrhages, a ≤ 20% increase in volume compared to baseline at both 1- and 12-hours post-infusion was “excellent” hemostasis, while a ≤35% increase compared to baseline at 12 hours was considered “good.” • The scoring for subarachnoid and subdural bleeds was similar, except maximal hematoma thickness was used		• CT scan data did not report bleed sizes to estimate volume changes, so hemostasis within 12 hours of andexanet alfa administration after 4F-PCC could not be established based on ANNEXA-4 criteria • Andexanet was given before stability head CT scans in 77.78% cases • In 33.33% of cases, repeat scans showed continued bleeding directly after andexanet administration, which could approximate 66.67% efficacy after andexanet administration
Safety outcomes	• Death occurred in 49 (14%) patients within 30 days, 35 of cardiovascular causes, 12 of non-cardiovascular causes, and 2 of unknown causes • 10% incidence of thromosis: 11 within 5 days of andexanet therapy, 11 within 6 and 14 days, and 12 within 15 to 30 days • 8 of these patients had their thrombotic event after restarting anticoagulation while inpatient		• This retrospective case series had a high mortality rate of 55.56% within 30 days but did not exclude patients with a low likelihood of survival • 55.56% of patients in this series experienced a thrombotic event • Five patients in this case series started anticoagulation as prophylaxis while inpatient, with three of these five experiencing thrombosis despite prophylaxis

In this case series, many instances of andexanet alfa administration after 4F-PCC were made without additional CT scans, which, while reflective of clinical practice, may not be best practice if the option for additional data is available. While not all patients had repeat head CT scans between the administration of 4F-PCC and andexanet alfa, the two patients who had a CT scan before andexanet alfa administration and therefore between reversal agent administration showed worsening or evolving hemorrhage. Theoretically, the administration of an additional agent may have been appropriate. For the remaining seven cases where andexanet alfa was given prior to a repeat head CT, it is not clear whether 4F-PCC helped with bleed stabilization. However, there were three cases (33.33%) where the bleed increased or evolved even after andexanet administration. Though it cannot be elucidated whether andexanet, 4F-PCC, or even additional blood products played a larger role in ICH stabilization, six cases (66.67%) showed potential hemostasis.

Compared to ANNEXA-4, this cohort did not check anti-Xa levels due to lack of availability; however, this is not standard of care in most institutions and does not align with bleeding and thrombosis rates. Though this was the primary outcome of ANNEXA-4 and only patients with anti-Xa activity ≥75 ng/mL were included in the efficacy analysis, as specifically written in the study, “there was no significant relationship between hemostatic efficacy and a reduction in anti-factor Xa activity during andexanet treatment.” Though the ANNEXA-4 primary outcome was assessed using anti-Xa levels, which have not been standardized to specific rates of bleeding or thrombosis, anti-Xa dependent efficacy was not assessed in this case series. No patients had anti-Xa levels drawn prior to andexanet alfa administration and after post-4F-PCC administration, highlighting the difficulty in determining appropriate patients to receive andexanet alfa, particularly after receiving an additional thrombotic agent.

This institution did not have thromboelastography available to guide factor or blood product repletion, leading to difficulties in decision making where there was missing data. Apixaban or rivaroxaban assays or anti-Xa levels are not routinely available in all institutions, and results are often delayed relative to the urgent timing of reversal agent administration. Because of the rebound of anti-Xa activity after andexanet alfa cessation and due to the lack of established relationship between hemostatic efficacy and anti-Xa activity, laboratory results alone should not guide decision making on andexanet alfa or 4F-PCC administration. In situations where thromboelastography is available, it can be utilized to guide need for specific factors or blood products, decreasing the possibility of administering a potentially unnecessary product (such as more andexanet alfa or 4F-PCC) that could increase risk of adverse effects. In situations where anti-Xa assays are available, they can be utilized to see if there is still DOAC activity after 4F-PCC administration.

Due to the nonspecific nature of 4F-PCC providing additional clotting factors, thrombotic risk remains a concern given limited data in its use for patients on oral factor Xa inhibitors. Based on the half-life of coagulation factors contained in pro-hemostatic agents such as 4F-PCC, a patient could be at risk for thromboembolic events for up to 14 days after administration.^35^ Meanwhile, andexanet alfa is associated with a prothrombotic state secondary to its inhibition of tissue factor pathway inhibitor activity and increase in thrombin generation.^36^ As shown in retrospective data, clotting risk increases more when 4F-PCC is used in combination with andexanet alfa, with the theoretical clotting concern mainly coming from 4F-PCC but exacerbated by andexanet alfa. Prior studies have found that most thrombotic events occur after about six days, and this case series found the same incidence and timeline.^12,27^ Still, it is difficult to determine whether the thromboses seen in this case were due to the combination of 4F-PCC with andexanet, either agent independently, or because anticoagulation was removed from patients already at high risk of a thrombotic event.

There did not appear to be a correlation between the dose of 4F-PCC received and the risk of thrombosis, with doses ranging from 17 to 72 units/kg. This is multifactorial, however, as the patients in this case series had different comorbidities and timing between doses, so correlation cannot fully be ascertained. This analysis found that five patients experienced thrombosis (55.56%) within 30 days of using the combination therapy, which aligns with previously described thrombotic rates in patients using this combination of therapy but is higher than reports of thrombosis in use of andexanet or 4F-PCC alone. Barra *et al.* reported three of 18 ICH patients (16.7%) and ANNEXA-4 reported 34 of 352 patients (10%) experienced thromboembolism during 30 days of observation after anticoagulation reversal with just andexanet alfa.^12,16^ Liu et al. reported a thrombosis incidence similar to this analysis (40%) and also combined previous data to find an incidence of in-hospital thromboembolism of 35% based on eight of 23 cases.^28^ This study’s data combined with that of Liu *et al*. and Bradshaw et al. shows a thromboembolism incidence of 15 cases in 37 total cases, or 40.54%. Unfortunately, the data in other case reports were not easily distinguished by neurologic bleed compared to bleeds of other locations, so this incidence of thromboembolism is an approximation and rates in only ICH patients cannot be fully estimated without further study.

Patients who survived to discharge were more likely to have received less than the standard weight-based dose of 50 units/kg: three of nine patients (33.33%) who received less than 50 units/kg were discharged to a rehabilitation center or skilled nursing home. This is hypothesis-generating as to whether there is potential benefit to the combination of andexanet alfa administration after 4F-PCC only in patients who were not optimally dosed on 4F-PCC originally. There did not appear to be a correlation between worse outcomes and the time between reversal agents. Patients who survived to discharge ranged from about 1 hour to about 9 hours between doses. Patients who died during admission received the doses anywhere from 1 minute apart to about 6 hours apart.

There are several limitations to this case series. First, it had a small patient population and was from one institution only, limiting the generalizability of this information to other patient populations. Next, there may have been other factors present during the real-time clinical scenario that led to the decisions being made, as most cases featured administration of andexanet alfa without further scans or documented patient deterioration. While most cases had stable intracranial bleeding after administration of both agents, it cannot be determined whether the initial 4F-PCC dose stabilized the bleed rather than the addition of andexanet alfa, and patients who received additional blood products have added confounders. Additionally, it is unclear whether the dosing of 4F-PCC may have impacted the choice to use andexanet alfa or the incidence of thromboembolism. Further, most patients had low baseline SOFA scores but multifocal high-risk ICHs and SAHs, which is not the same study population as in the original ANNEXA-4 trial or in previous retrospective reviews. Analysis of initial CT impressions was not re-done for this case series, based on the fact that management had been based on those impressions. Additionally, a change in neurologic exam could have prompted the need for andexanet administration, but this not objectively easy to collect. Finally, this analysis had a very high mortality rate, so discovering the factors that may account for this difference is difficult.

Data on co-administration of 4F-PCC and andexanet alfa are sparse. Prior case reports that reported concomitant use of andexanet and 4F-PCC included all patients regardless of bleed location, and data could not be parsed as to specific ICH outcomes. In this cohort, many factors played a role in the possible outcomes, such as comorbidities, potential changes in action if factor Xa levels were available, inconsistent repeat CT scans, and timing of the second agent given. However, this mirrors clinical practice, and, to date, this is the only retrospective series looking at combined use of 4F-PCC and andexanet alfa in only nontraumatic neurocritical care patients with ICH, and there is still room for further research to elucidate the relationship between these two agents in clinical practice.

At this time, with the limited data available, high rates of thrombosis, and lower rates of bleed stability, use of the combination of 4F-PCC and andexanet alfa should not be considered without weighing risks and benefits. Risks of the combination include high rates of thrombosis, and patients on anticoagulation are already at considerable risk of thrombosis at baseline. Benefits of the combination include the possibility of halting further bleeding in patients who had continued worsening bleeding despite administration of a reversal agent. Patients with higher risk for thrombosis, such as those with multiple indications for anticoagulation, will likely be riskier candidates for 4F-PCC and andexanet alfa combination therapy. When utilizing the combination, consideration should be made to use the lower andexanet alfa dose, as the one patient in this case series who received the inappropriate high dose had an embolism and died.

Based on these cases, several changes to institutional practice can be considered. If the patient has not experienced bleed stability and is clinically worsening, foremost, hematology should be involved in cases where a second agent is being considered. If possible, a repeat CT scan is helpful to determine the need for a repeat dose. If the patient had a lower initial 4F-PCC dose, there is more room to consider use of additional 4F-PCC or andexanet alfa. Additionally, if the second agent being considered is andexanet alfa, anti-Xa activity can be measured either using agent-specific assays or by utilizing unfractionated heparin anti-Xa assays based on institutionaly availability and timeliness. Though heparin anti-Xa assays will not fully correlate with apixaban or rivaraxoaban anti-Xa assays, this will show if there is still activity after 4F-PCC was administered. If andexanet alfa is the first agent used, anti-Xa monitoring would not be accurate. This should not guide decisions alone but should serve as one of the factors in the decision. Thromboelastography can also be considered in these situations for ensuring appropriate agents or blood products are utilized overall. Finally, the risk of thrombosis in this case series and in the pooled data of prior cases is high, so patients at particularly high risk of thrombosis, such as those with a persistent history of clots, cancer history, or a factor deficiency, may not find additional benefit from a second agent.

## Conclusion

This retrospective analysis of patients receiving the combination of 4F-PCC and andexanet alfa reveals the possible risks and benefits of co-administering both agents in nontraumatic ICH patients. With an incidence of thromboembolism of 55.56% and an incidence of hemostasis of 66.67% in this cohort alone, the efficacy and safety of using these agents in combination for reversal of nontraumatic ICH remains unclear. Real-world application of 4F-PCC and andexanet alfa therapy in combination is more diverse and unpredictable, requiring further study.
